# Opacités pulmonaires excavées persistantes au cours d'une granulomatose avec polyangéite: penser à l'aspergillose

**DOI:** 10.11604/pamj.2015.20.275.6422

**Published:** 2015-03-20

**Authors:** Madiha Mahfoudhi, Sami Turki

**Affiliations:** 1 Service de Médecine Interne A, Hôpital Charles Nicolle, Tunis, Tunisie

**Keywords:** Granulomatose avec polyangéite, aspergillose pulmonaire, immunosuppresseur, Granulomatosis with polyangiitis, pulmonary aspergillosis, immunosuppressive

## Image en medicine

La granulomatose avec polyangéite peut se compliquer d'infections parfois graves vu la prescription d'une corticothérapie au long cours et de traitements immunosuppresseurs. Patient âgé de 50 ans admis pour une hémoptysie récidivante de faible abondance, une toux, un purpura vasculaire et une obstruction nasale. L'examen biologique a révélé un syndrome inflammatoire biologique avec une anémie normochrome normocytaire (hémoglobine:11 g/dl). La protéinurie par 24 heures était négative. Le scanner thoracique a objectivé des opacités pulmonaires excavées. Le scanner du massif facial a montré un comblement des sinus maxillaires. La biopsie bronchique a révélé un aspect de vascularite granulomateuse. Le bilan immunologique a révélé des c ANCA fortement positifs. Le diagnostic d'une granulomatose avec polyangéite a été retenu. Il a été traité par une corticothérapie associée à des boli de cyclophosphamide et une antibiothérapie à base de sulfamethoxazole et trimethoprim. L’évolution était marquée par la disparition des signes cutanés et ORL, ainsi que le syndrome inflammatoire biologique. Cependant les signes respiratoires ne se sont pas améliorés. La tomodensitométrie thoracique a montré une augmentation de la taille des nodules pulmonaires excavés ayant des diamètres allant de 12 mm à 25 mm. Des infections notamment une tuberculose, une pneumocystose ou une aspergillose pulmonaire ont été évoquées. La sérologie aspergillaire était positive. La culture sur milieu de Sabauraud du liquide de lavage broncho-alvéolaire a isolé un Aspergillus fumigatus. Un traitement antifongique de type voriconazole a été prescrit avec une bonne évolution des signes respiratoires aussi bien cliniques que radiologiques.

**Figure 1:**
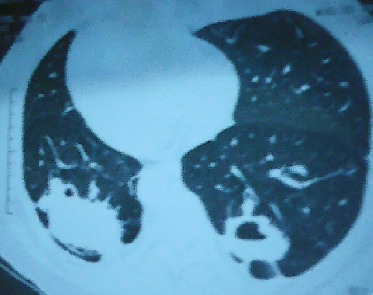
TDM thoracique: multiples nodules excavés des deux champs pulmonaires ayant des diamètres allant de 12 mm à 25 mm

